# The Case for the Eight Hours Day
*See "Eight Hours for Work." By John Rae, M.A., Author of "Contemporary Socialism." (London and New York: Macmillan and Co. 1894.)


**Published:** 1895-02-02

**Authors:** 


### Modern Sociology.

### THE CASE FOR THE EIGHT HOURS DAY.*

AMONG the many panaceas recommended for freeing society from that anxious burden, the unemployed, none is more popular, both with working men and those who are called by their friends labour leaders, and by their enemies agitators, than the shortening of the working day in order to employ more hands to produce the same output of work. But one may question if the working men and their friends are right in assuming that to make the hours of work shorter would, under existing circumstances, necessitate the employment of more workers. It was not so in the past, when the hours of labour were reduced at a bound from twelve, thirteen, or fourteen to ten, and without going to the *reductio ad absurdum* of saying that the less a man works the more he will produce, it may be doubted if a further shortening of the working day to eight hours will greatly, if at all, decrease production.

Long hours of work came in with machinery and the factory system. Masters who had set up expensive machinery hated to see it standing idle, and made their work hours as long as human power could stand, or even longer. For the workers, though bribed by higher wages than their fathers had ever earned, cried out at last for rest. Philanthropists demanded the same on their behalf; the demand was made and granted in obedience to moral, not economic laws, but the economic result was startling. Production did not decrease, as the masters had feared; wages were not lowered, as the workmen had expected. In some cases there was even an increase of production, in others the first few weeks saw a diminution, but this was soon repaired and often changed to increase; in none was there any permanent loss of production. Some small part of the credit of this may have been due to improvements in machinery, but it was the human machines that were the most improved. The "hands" began to employ their heads; they worked with more precision, more attention, more intelligence. Even with piece-workers this was the case. We naturally assume that a man on piece-work will work as hard as he can, but this "can" is a variable quantity. A man can do more and better work when he is fresh than when he is tired, and even piece-workers found the shorter work hours profitable. It is impossible to give detailed statistics here, but the records of every country bear the same testimony, crystallised in the surprised exclamation of one employer, "We used to say that the last hour made our profits; we find that it was the last hour that ate up our profits."

But can the principle be carried further with the same results? As legal compulsion has not yet come in, the statistics obtainable are comparatively small, but the experiment has been tried and has succeeded. Mr. W. Allan, M.P., has, since 1892, been working eight hours in his engineering works in Sunderland, and his testimony is this: "Paradoxical as it may seem, I get fully more work out than formerly; in fact, I am surprised at how the work is going ahead, having believed, like so many employers, that there would be a corresponding decrease in output." Mr. Harrison, manager to Mr. Allan, mentioned a detail still more surprising: "A certain quantity of work used to be turned out by each machine under the nine hours system. Incredible as it may seem, the same amount of work is turned out by the same machine while worked for eight hours only." Has the machine been improved? No; but, Mr. Harrison said, the men wasted less time and worked with more energy. About five years ago Messrs. S. and H. Johnson, of Stratford, London, inaugurated the eight hours system. Their experience is the same as Mr. Allan's. The amount of work turned out is the same, and there is no increase in the cost of production. Indeed, there are some savings in gas, coal, &c., which would go to counter-balance any loss incurred in paying the same wages for a shorter day's work. But it is not on this that the success of the experiment has depended. By changing the hours from six to five to eight to five, they start after the men have breakfasted, and thus have only one break for meals. Formerly the men coming fasting to their work were languid till the breakfast hour. This languor has now vanished. A quarter of an hour, too, is saved daily by this abolition of the breakfast hour, as it took that time to prepare for going away before and starting work after breakfast. Elsewhere that the same experiment has been tried the same results have followed. Lord Brassey gives an instance as applied to the building trade: "During the construction of the Trent Valley line of railway, immense efforts were made to complete the work in the shortest possible time, and in order to expedite to the utmost degree the completion of the station at Atherstone, two shifts of men were employed in the building, each of them working eight hours a day. It was found that each shift, although working for only eight hours, did more work in a day than other men employed for the full number of hours which at that time constituted a day's work, viz., ten hours a day." A German manufacturer, Herr Freese, window-blind maker, who has adopted the eight hours system, and whose hands are mostly paid by the piece, records that some of these earned less and some more, but the general average of wages was higher than under the old system, and in no case was the loss proportionate to the diminution of working hours.

Of course, there are people whom not even statistics will move from their conviction that the old ways are the best. Mr. Seaton, managing director of the Hull Engineering Company, is one of these, and he poured scorn on Mr. Allan's experiment in his evidence before the Labour Commission. All that Mr. Allan has proved, he says, "is that by giving the men shorter hours, and encouraging them in various ways, he has got them to work harder for him than they would for me." If that be so, why does not Mr. Seaton follow Mr. Allan's example? This, which he states so scornfully, is just the case for the eight hours day. Certainly everywhere that it has been tried the system has been successful in every point but the one which its most ardent advocates have at heart—it will not diminish the output per man; it will not relieve us of the unemployed.

*See "Eight Hours for Work." By JOHN RAE, M.A., Author of "Contemporary Socialism." (London and New York: Macmillan and Co. 1894.)

